# Protective Effect of Pomegranate on Oxidative Stress and Inflammatory Response Induced by 5-Fluorouracil in Human Keratinocytes

**DOI:** 10.3390/antiox10020203

**Published:** 2021-01-30

**Authors:** Shara Francesca Rapa, Giorgia Magliocca, Giacomo Pepe, Giuseppina Amodio, Giuseppina Autore, Pietro Campiglia, Stefania Marzocco

**Affiliations:** 1 Department of Pharmacy, University of Salerno, Via Giovanni Paolo II 132, 84084 Fisciano, SA, Italy; srapa@unisa.it (S.F.R.); g.magliocca1@studenti.unisa.it (G.M.); gipepe@unisa.it (G.P.); autore@unisa.it (G.A.); pcampiglia@unisa.it (P.C.); 2 Department of Medicine, Surgery and Dentistry “Scuola Medica Salernitana”, University of Salerno, Via Salvador Allende, 84081 Baronissi, SA, Italy; gamodio@unisa.it

**Keywords:** 5-Fluorouracil, human keratinocytes, pomegranate juice extract, oxidative stress, inflammation, apoptosis, wound repair

## Abstract

5-Fluorouracil (5-FU) is a pyrimidine analogue used as an antineoplastic agent to treat multiple solid tumors. Despite its use and efficacy, it also has important side effects in healthy cells, including skin reactions, related to its pro-oxidant and pro-inflammatory potential. Although there are numerous remedies for chemotherapy-induced skin reactions, the efficacy of these treatments remains limited. In this study we focused on the effects of pomegranate (*Punica granatum* L.) juice extract (PPJE) on the oxidative and inflammatory state in 5-FU-treated human skin keratinocytes (HaCaT). The obtained results showed that PPJE significantly inhibited reactive oxygen species release and increased the cellular antioxidant response, as indicated by the increased expression of cytoprotective enzymes, such as heme oxygenase-1 and NAD(P)H dehydrogenase [quinone] 1. In these experimental conditions, PPJE also inhibited nitrotyrosine formation and 5-FU-induced inflammatory response, as indicated by the reduced cytokine level release. Moreover, PPJE inhibited nuclear translocation of p65-NF-κB, a key factor regulating the inflammatory response. In 5-FU-treated HaCaT cells PPJE also inhibited apoptosis and promoted wound repair. These results suggest a potential use of PPJE as an adjuvant in the treatment of the oxidative and inflammatory state that characterizes chemotherapy-induced skin side effects.

## 1. Introduction

Chemotherapeutic treatment is associated with severe toxic effects that vary with the dose and the patient, and lead sometimes to chemotherapy discontinuation which adversely affects the prognosis and eventually reduces the survival rates of patients. The therapies target specifically not only cancer cells but also normal proliferating cells, causing inhibition of cell proliferation, genomic instability, overproduction of different reactive toxic products and mediators and necrosis, leading to severe toxic effects and complications. 5-Fluorouracil (5-FU), a pyrimidine analogue, is among the most widely used drugs in the treatment of many types of cancer. Like most chemotherapeutic agents, 5-FU does not have a selective action targeting cancer cells and this leads to a number of serious side effects [1]. Different complications are associated with 5-FU therapy and among the most common complications are gastrointestinal effects and dermatitis [2,3,4,5,6,7,8]. In particular, dermatitis is a frequent side effect of chemotherapy and radiotherapy treatment in cancer patients. Dermatitis arises as the beginning of erythema, swelling, an acneiform rash, severe pruritus, xerosis cutis, skin wounds and inflammatory ulcers, which can lead to chronic inflammation, necrosis, fibrosis and increased risk of infections in patients [9,10,11]. 5-FU may rapidly harm dividing immature keratinocytes, as well as dividing stem cells [12,13,14] and the basal cell layer of epithelium can be directly damaged leading to the loss of the renewal capacity of the epithelium, which may result in ulceration [15].

Among the etiological factors that can determine the pathogenesis of toxic side effects induced by chemotherapy treatments, oxidative stress and inflammation are important [16,17]. In particular, chemotherapy drugs are responsible for an excessive production of radical oxygen species (ROS) and inflammatory mediators that trigger pathological mechanisms of cell damage, altering the normal physiological parameters of the cells [18,19]. In particular, ROS levels are also increased after 5-FU treatment and its induction is also critical to promote both inflammation and drug-mediated apoptotic cell death [20]. Therefore, inhibiting or limiting ROS production and inflammation may help in the treatment of dermatitis in cancer patients.

Although various types of therapies have been introduced for the prevention and treatment of chemotherapy-induced dermatitis, the efficacy of these treatments remains limited, and thus researchers have a great interest in finding new therapeutic strategies also in the field of natural products.

Pomegranate (*Punica granatum* L.) was one of the first domesticated fruits to be cultivated and has also gained considerable recognition as a functional food in the modern era. A large body of literature has highlighted the health-promoting activities of pomegranate juice and fruit extracts. Pomegranate has been used to expel parasites [21], its seeds and fruit peels to treat diarrhea [22,23], its plant flowers to manage diabetes [24], its tree barks and roots to end bleeding and heal ulcers, and its leaves to manage inflammation and handle digestive system disorders [25]. The ingredients of pomegranate also show a chemopreventive role through the inactivation and activation of different cell signaling pathways [26,27] and their effect in counteracting some chemotherapy-induced side effects, such as nephrotoxicity, hepatotoxicity and intestinal mucositis, have also been reported [28,29]. Considering the properties of the polyphenols present in pomegranate juice extract (PPJE), in this study we evaluated its effect on oxidative stress, apoptosis and cellular migration in a human keratinocyte cell line (HaCaT) treated with 5-FU.

## 2. Materials and Methods

### 2.1. Reagents

Unless stated otherwise, all reagents and compounds were purchased from Sigma Chemicals Company (Sigma, Milan, Italy).

### 2.2. Sample Preparation

*Punica granatum* L. fruits were purchased in a local store in Fisciano (SA). Fruits were manually washed and peeled, then the arils were squeezed with an electric homogenizer. The juice obtained was centrifuged and then the supernatant purified by solid phase extraction.

The polyphenolic extract (PPJE) was eluted with methanol, evaporated to dryness under vacuum at 40 °C in a rotary evaporator, and finally dissolved in methanol, filtered and then analyzed by LC × LC system coupled online to IT-TOF instrument equipped with an electrospray source (ESI). HILIC × RP-MS/MS parameters and the full list of identified polyphenolic compounds are in complete agreement with data previously reported by us [29].

### 2.3. Cell Culture

The immortal keratinocyte (HaCaT) cell line, derived from normal adult human skin, was purchased from CLS Cell Lines Service GmbH (Eppelheim, Germany—accession number 300493) [30]. HaCaT cells are routinely maintained in the presence of Dulbecco’s modified Eagle’s medium (DMEM) containing 10% (*v*/*v*) fetal bovine serum (FBS) and 1% (*v/v*) penicillin-streptomycin. Cells were grown at 37 °C in a humidified atmosphere of 5% CO_2_/ 95% air.

### 2.4. Cellular Treatment

In order to estimate the antioxidant and anti-inflammatory potential at skin level of pomegranate juice extract (PPJE) on HaCaT during chemotherapy treatment, cells were plated and, after adhesion, treated with PPJE (1.25–10 µg/mL) alone, for 1 h, and then simultaneously with 5-FU (10 µg/mL), for different times based on the mediators evaluated. Details regarding sample preparation and the qualitative and quantitative profile of the PPJE are reported above [29].

### 2.5. Measurement of Intracellular ROS Release

ROS levels were evaluated by means of the probe 2′,7′-dichlorofluorescin-diacetate (H_2_DCF-DA) [31]. HaCaT cells were plated in 24-well plates (8.0 × 10^3^ cells/well) and, after adhesion, were treated as previously described, for 24 h. The cells were then collected, washed with phosphate buffered saline (PBS), and then incubated in PBS containing H_2_DCF-DA (10 μM). After 15 min at 37 °C, cell fluorescence was evaluated using a fluorescence-activated cell sorter (FACSscan; Becton Dickinson, Franklin Lakes, NJ, USA) and analyzed with Cell Quest software (version 4; Becton Dickinson, Milan, Italy).

### 2.6. Evaluation of HO-1, NQO1, Bax and Bcl-2 Expression and Nitrotyrosine Formation by Flow Cytometry

After the cellular treatment, HaCaT were collected, washed with PBS, then incubated in fixing solution for 20 min and then in Fix Perm solution for 30 min. Anti-heme oxygenase-1 (HO-1; Santa Cruz Biotechnologies, Dallas, TX, USA), anti-NAD(P)H Quinone Dehydrogenase 1 (NQO-1; Santa Cruz Biotechnologies), anti-Bax (Santa Cruz Biotechnologies, Dallas, TX, USA), anti-Bcl-2 (Santa Cruz Biotechnologies, Dallas, TX, USA) or anti-nitrotyrosine (Merck Millipore, Milan, Italy) antibodies were then added for 1 h. The secondary antibody was added to HaCaT cells in fixing solution and cell fluorescence was then evaluated by a fluorescence-activated cell sorter (FACSscan; Becton Dickinson, Milan, Italy) and then elaborated by Cell Quest software (version 4; Becton Dickinson, Milan, Italy) [32].

### 2.7. TNF-α, IL-6 and IL-1β Levels

Following the cellular treatment described above, the cytokine levels were measured in the supernatants of the treated HaCaT cells, through the use of an enzyme-linked immunosorbent assay (ELISA), according to the manufacturer’s instructions. Data were expressed as pg/mL of cytokine release [33].

### 2.8. Quantitative Imaging of NF-κB Nuclear Translocation

HaCat cells were seeded on glass cover slips and treated with PJJE (2.5–10 μg/mL) and 5-FU, as described above, for 4 h. Cells seeded on glass cover slips were then washed with PBS, fixed in PBS 4% paraformaldehyde (PFA) and processed for immunofluorescence as previously described [34,35]. In particular, after fixation cells were quenched with 50 mM NH_4_Cl. Then, cells were permeabilized 5 min with PBS 0.1% Triton and blocked for 30 min with PBS containing 0.5% bovine serum albumin (BSA) and 50 mM NH_4_Cl. Cells were immunostained with the primary antibody mouse monoclonal anti-NF-κB p65 (Santa Cruz Biotechnology: sc-8008). Primary antibody was detected with Alexa 488 (Jackson Immuno Research). Nuclei were counterstained with 2 μg/mL of the nuclear stain DAPI for 5 min. For each image, z-stacks centered around the plane with maximum DAPI (4′,6-Diamidino-2-phenylindole dihydrochloride) staining were acquired on a laser scanning confocal microscope (TCS SP5; Leica MicroSystems) equipped with a plan Apo 63X, NA 1.4 (Carl Zeiss, White Plains, NY 10601 United States) soil immersion objective lens taking care to use the same parameters and to remain below saturation. Quantitative analysis was performed by the Image software on a minimum of 50 cells for each sample. Automatic thresholding was applied and binary image masks were created of NF-κB and DAPI positive staining to define regions of interest (ROI) for analysis [36,37]. The DAPI staining mask was used to define the nuclear ROI. Using the image calculator, the DAPI mask was subtracted from the NF-κB mask to create a staining mask defining the cytoplasmic ROI. Each of these ROI masks were then applied to the original NF-κB staining images to separate nuclear and cytoplasmic staining within each field. Mean intensity of fluorescence was measured in the defined ROI to calculate nuclear/cytoplasmic ratio.

### 2.9. Propidium Iodide Assay

The percentage of hypodiploid nuclei was analyzed using propidium iodide (PI) staining by flow cytometry [38]. Briefly, HaCaT cells (8.0 × 10^3^ cells/well) were grown in 24-well plates and allowed to adhere. Thereafter cells were exposed to PPJE (1.25–10 μg/mL) in combination with 5-FU (10 μg/mL), for a further 24 h. Subsequently, after removing the cell medium and washing the cells with PBS, the treated HaCaT cells were suspended in 500 µL of a solution consisting of 0.1% (*w*/*v*) sodium citrate and 50 µg/mL PI. At the same time, the cell medium was centrifuged and the cell pellet was added to the cell suspension in order to evaluate both live and dead cells. After incubation for 30 min, al 4 °C and in the dark, hypodiploid nuclei were analyzed with FACScan flow cytometer (Becton Dickinson, Franklin Lakes, NJ, USA) and analyzed with Cell Quest software (ver-sion 4; Becton Dickinson, Milan, Italy).

### 2.10. Wound Healing Assay

For the wound healing assay, HaCaT cells were plated in 24-well plates (8.0 × 10^3^ cells/well) and allowed to adhere. Then, a wound was produced at the center of the cell monolayer, scraping the bottom with a sterile p10 pipette. The cells were washed with PBS and incubated with cell medium alone or with 5-FU alone (10 μg/mL) or with PPJE (2.5–5 μg/mL) plus 5-FU (10 μg/mL) in a balanced incubation chamber (Integrated Live Cell Workstation Leica AF-6000 LX) with a controlled environment (37 °C; 5% *v*/*v* CO_2_). A 10× phase contrast objective was used to record cell movements with a frequency of acquisition of 10 min. At the end of the acquisition, three different positions were considered for each wound, and the movement of ten randomly selected cells was analyzed for each wound. Cell migration distances were calculated from time 0 to the chosen time points, using the Leica ASF software. Statistical evaluation was performed with the GraphPad Prism 4 software (GraphPad, San Diego, CA, USA) [39].

### 2.11. Data Analysis and Statistical Evaluation

Data are shown as mean ± mean standard error (s.e.m.), of at least three independent experiments, performed in triplicate. Statistical evaluation was performed using GraphPad Prism 4 software (San Diego, CA, USA), through analysis of variance test and Bonferroni’s test for multiple comparisons A P-value lower than 0.05 was considered significant.

## 3. Results

### 3.1. PPJE Inhibited ROS Release and Increased HO-1 and NQO1 Expression in 5-FU-Treated HaCaT Cells

**Figure 1:**
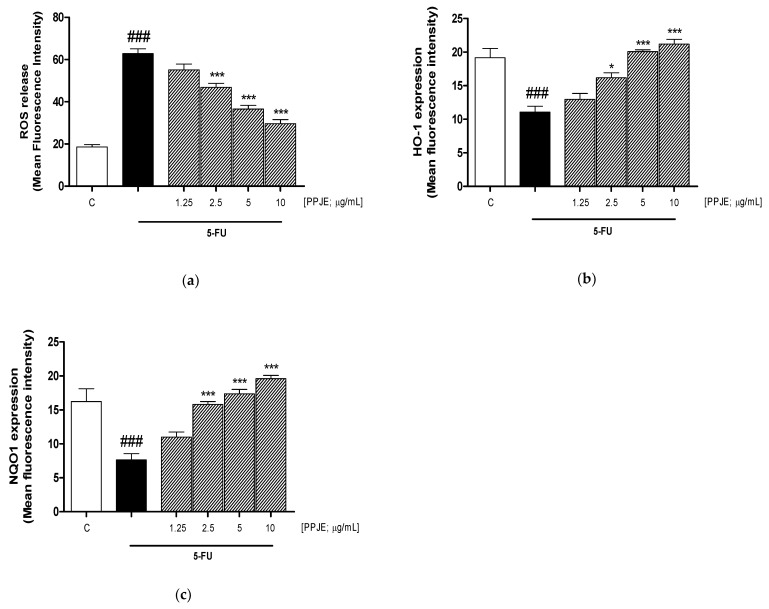
Effect of pomegranate juice extract (PPJE; 1.25–10 µg/mL) on intracellular ROS (reactive oxygen species) release (**a**), evaluated by means of the probe 2′,7′-dichlorofluorescin-diacetate (H_2_DCF-DA), and HO-1 (**b**) and NQO1 (**c**) expression, evaluated by cytofluorimetric technique, on HaCaT cells treated with 5-fluorouracil (5-FU; 10 µg/mL). Data are expressed as mean of fluorescence intensity. C denotes control group. ### denotes *p* < 0.001 vs. C; *** and * denote respectively *p* < 0.001 and *p* < 0.05 vs. 5-FU.

In order to investigate the antioxidant potential of PPJE, we evaluated its ability to reduce intracellular release of ROS, induced by 5-FU. Our results indicated that PPJE (1.25–10 µg/mL) significantly inhibited ROS release in 5-FU-treated (10 µg/mL) HaCaT cells at the three highest tested concentrations (*p* < 0.001 vs. 5-FU; Figure 1a).

Oxidative stress is triggered by the imbalance between the excessive production of ROS and the reduced antioxidant defense capabilities in cells. For this reason, we analyzed the expression of two antioxidant and cytoprotective enzymes, HO-1 and NQO1. Flow cytometric analysis demonstrated that PPJE (1.25–10 µg/mL) was able to significantly increase the expression of HO-1 (*p* < 0.05 vs. 5-FU; Figure 1b) and NQO1 at the three highest tested concentrations (*p* < 0.001 vs. 5-FU; Figure 1c) in HaCaT cells treated with 5-FU.

### 3.2. PPJE Reduced Nitrotyrosine Formation in 5-FU-Treated HaCaT Cells

**Figure 2:**
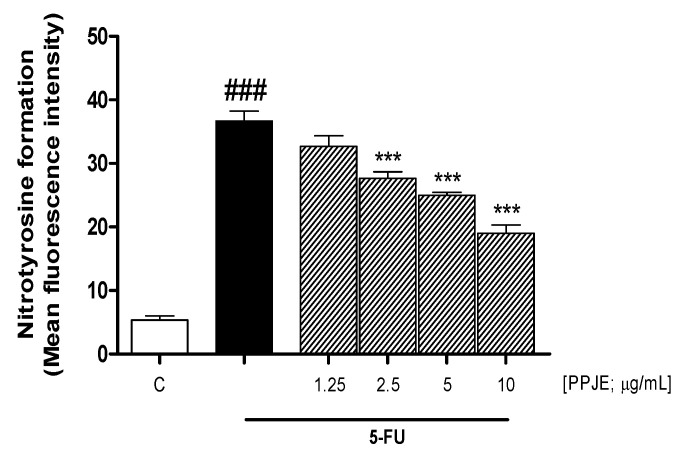
Effect of pomegranate juice extract (PPJE; 1.25–10 µg/mL) on nitrotyrosine formation, evaluated by cytofluorimetric technique, on HaCaT cells treated with 5-fluorouracil (5-FU; 10 µg/mL). Data are expressed as mean of fluorescence intensity. C denotes control group. ### denotes *p* < 0.001 vs. C; *** denotes *p* < 0.001 vs. 5-FU.

Nitrotyrosine is an important biomarker of oxidative stress, formed by the nitration of the tyrosine residues of proteins from reactive peroxynitrite species. The resulting nitration of protein residues can play an important role in the pathogenesis of inflammatory skin diseases. In this work we have shown that 5-FU significantly increased nitrotyrosine formation compared to untreated cells (*p* < 0.001 vs. C; Figure 2), but PPJE (1.25–10 µg/mL) reduced the formation of this biomarker in 5-FU-treated keratinocytes, significantly at 2.5, 5 and 10 µg/mL (*p* < 0.001 vs. 5-FU; Figure 2).

### 3.3. PPJE Reduced Cytokine Levels

**Figure 3:**
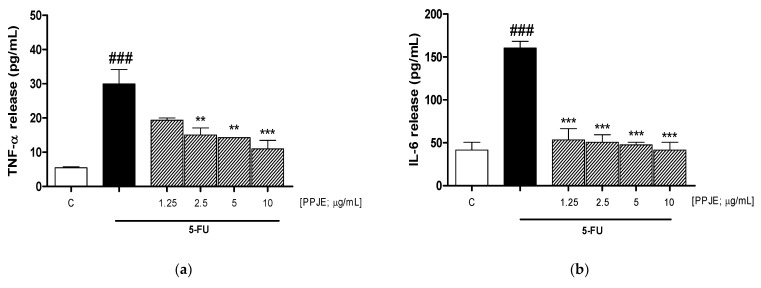

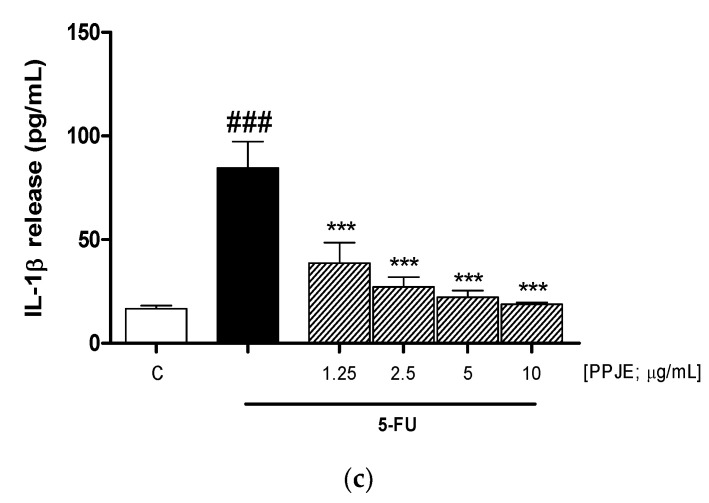
Effect of pomegranate juice extract (PPJE; 1.25–10 µg/mL) on TNF-α (tumor necrosis factor-α) (**a**), IL-6 (interleukin-6) (**b**) and IL-1β (interleukin-1β) (**c**), evaluated by ELISA assay, on HaCaT cells treated with 5-fluorouracil (5-FU; 10 µg/mL). Data are expressed as pg/mL of cytokine release. C denotes control group. ### denotes *p* < 0.001 vs. C; *** and ** denote respectively *p* < 0.001 and *p* < 0.01 vs. 5-FU.

A positive correlation exists between oxidative damage and release of pro-inflammatory cytokines. Moreover, scientific evidence has reported that the release of pro-inflammatory cytokines by cutaneous keratinocytes is implicated in various inflammatory skin diseases, also caused by 5-FU [15]. Using an ELISA assay, we evaluated the ability of PPJE to reduce pro-inflammatory cytokine levels in the supernatants of 5-FU-treated HaCaT cells. Our results showed that PPJE (1.25–10 µg/mL) significantly reduced the levels of tumor necrosis factor-α (TNF- α; 2.5–10 µg/mL; *p* < 0.01 vs. 5-FU; Figure 3a), interleukin-6 (IL-6; *p* < 0.001 vs. 5-FU; Figure 3b) and interleukin-1β (IL-1 β; *p* < 0.001 vs. 5-FU; Figure 3c) for all the tested concentrations in 5-FU-treated HaCaT cells.

### 3.4. PPJE Inhibited NF-κB Translocation in 5-FU Treated HaCaT Cells

**Figure 4:**
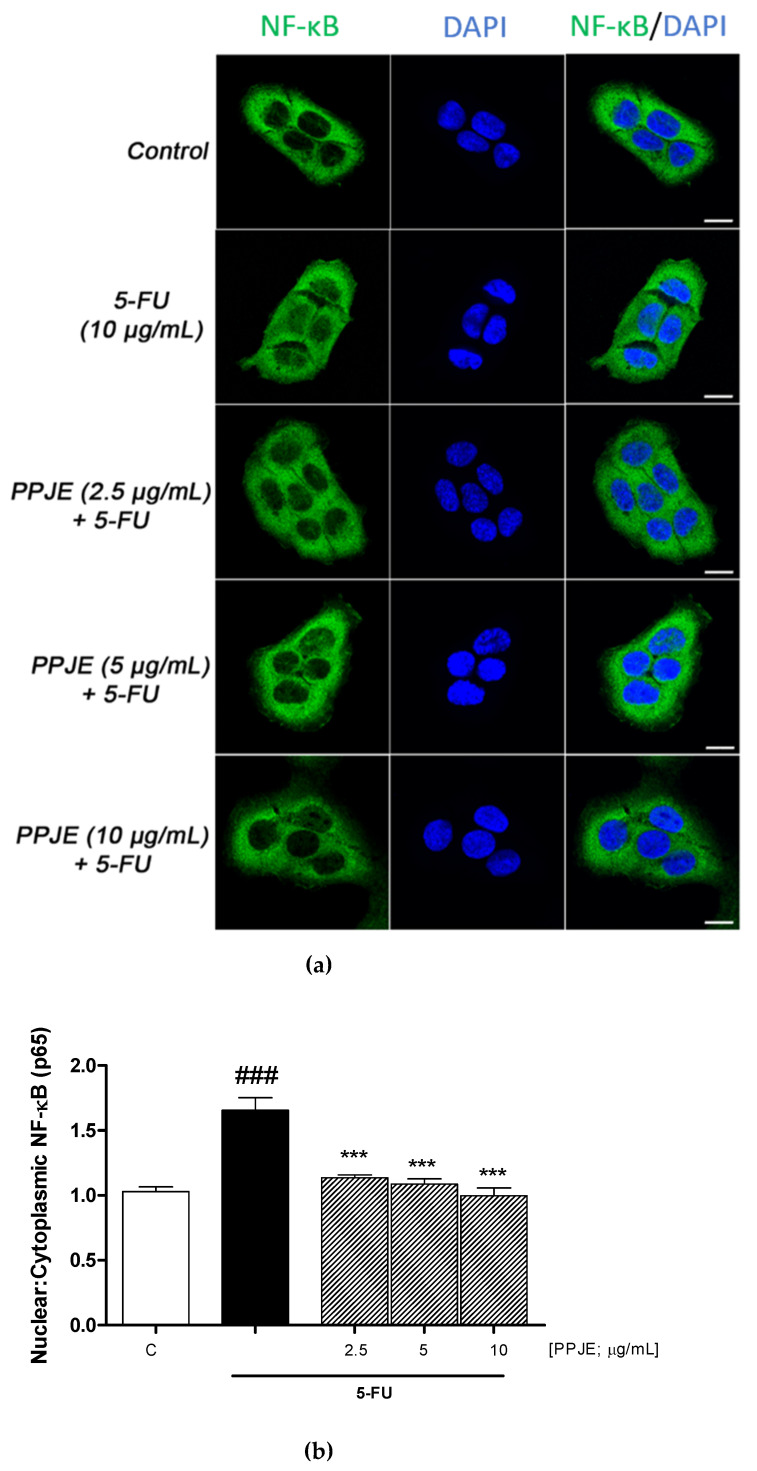
Immunofluorescence staining of NF-κB (**a**) and quantification of nuclear/cytoplasmic ratios of NF-κB staining (**b**) in HaCaT cells treated for 4 h with 5-FU (10 μg/mL) alone or in combination with increasing concentration of PPJE (2.5–10 μg/mL). (**b**) Histograms represent the mean ± s.e.m. of nuclear/cytoplasmic ratios of NF-κB staining calculated as described in methods section. C denotes control group. ### denotes *p* < 0.01 vs. C; *** denote *p* < 0.001 vs. 5-FU.

In order to analyze the effect of PPJE directly on NF-κB activation, we performed a quantitative immunofluorescence-imaging assay of the nuclear translocation of NF-κB in HaCaT cells exposed to 5-FU alone or in combination with PPJE (2.5–10 μg/mL). As expected, we found that in HaCaT cells the 5-FU treatment (10 μg/mL; 4 h) induced the cytoplasmic to nuclear translocation of NF-κB with a significant 1.7-fold increase in the nuclear/cytoplasmic ratio of NF-κB staining. Conversely, the treatment with PPJE significantly inhibited the nuclear translocation of NF-κB in response to 5-FU with a significant effect evident across the entire PPJE dose range, including the lower dose (*p* < 0.001 vs. 5-FU; Figure 4 a, b). These results demonstrate that PPJE, inhibiting NF-κB nuclear localization, could have a protective effect on HaCaT in the presence of 5-FU.

### 3.5. PPJE Reduced 5-FU-Induced Apoptosis in HaCaT Cells

**Figure 5:**
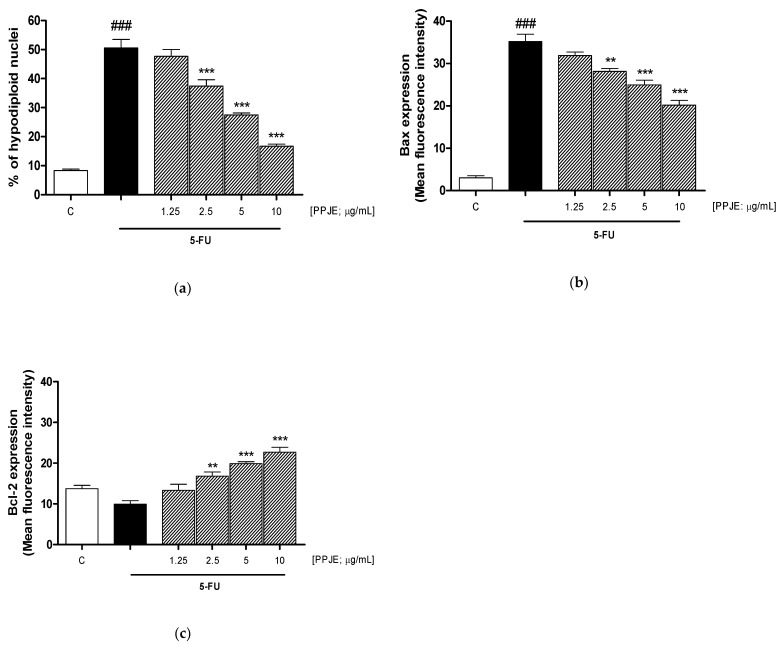
Effect of pomegranate juice extract (PPJE; 1.25–10 µg/mL) on apoptosis (**a**), evaluated by propidium iodide assay, and on Bax (**b**) and Bcl-2 (**c**), evaluated by cytofluorimetric technique, on HaCaT cells treated with 5-fluorouracil (5-FU; 10 µg/mL). Data are expressed as % of hypodiploid nuclei or as mean of fluorescence intensity. C denotes control group. ### denotes *p* < 0.001 vs. C; *** and ** denote respectively *p* < 0.001 and *p* < 0.01 vs. 5-FU.

5-FU induces apoptosis in human keratinocytes, which is closely related to an increase in oxidative stress markers, both as increased super anion levels and increased membrane lipid peroxidation [40]. To evaluate whether PPJE could negatively modulate apoptosis induced by 5-FU, we analyzed the percentage of hypodiploid nuclei, through the PI (propidium iodide) assay. Figure 5a shows that the tested extract (1.25–10 µg/mL) significantly reduced 5-FU-induced apoptosis in HaCaT cells (2.5–10 µg/mL; *p* < 0.001 vs. 5-FU; Figure 5a).

Interestingly, PPJE significantly reduced the expression of the pro-apoptotic protein Bax (*p* < 0.01 vs. 5-FU; Figure 5b) and increased the expression of the anti-apoptotic protein Bcl-2 (*p* < 0.01 vs. 5-FU; Figure 5c) at the three highest tested concentrations.

### 3.6. PPJE Promoted Cell Migration Following Wound

**Figure 6:**
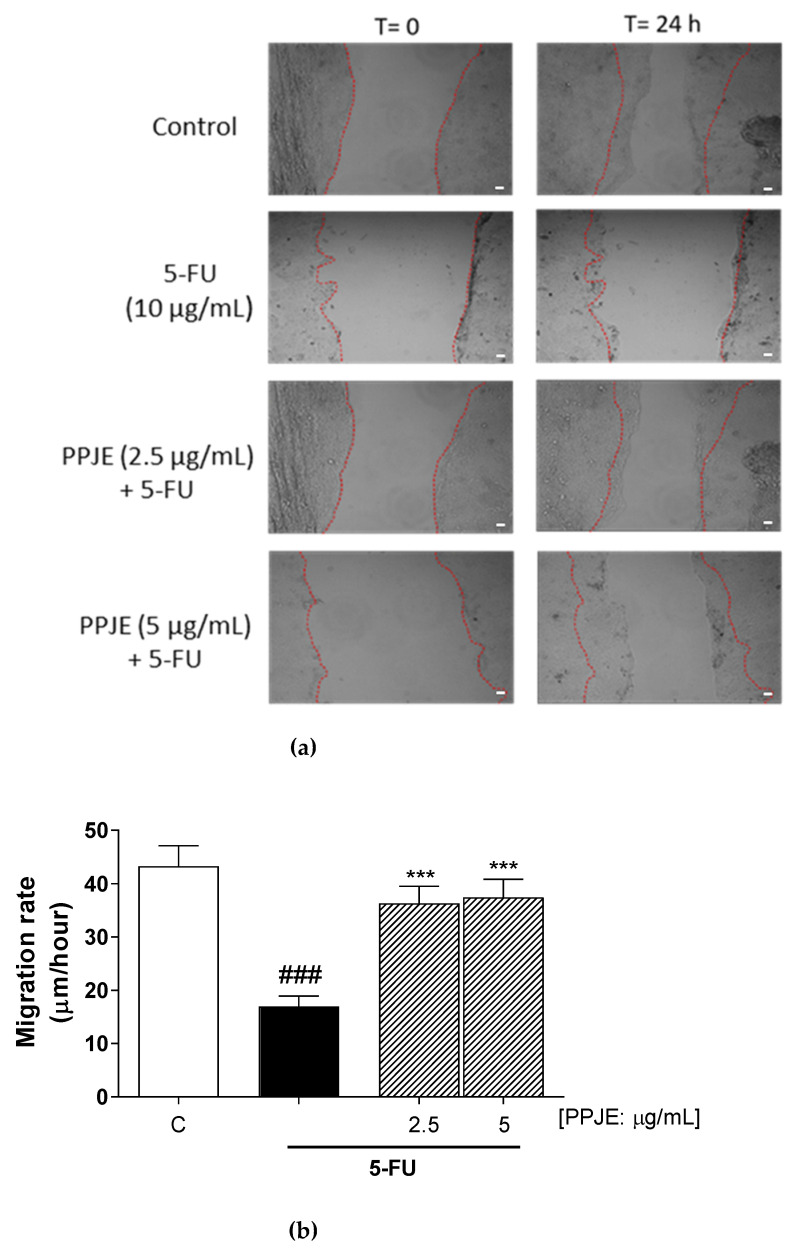
Effect of PPJE (2.5–5 µg/mL) on wound repair after a mechanical scratch in HaCaT cells treated with 5-fluorouracil. FU; 10 µg/mL); Bar = 150 μm (**a**), and the quantitative analysis expressed as HaCaT migration rate after 24 h (**b**). C denotes control group; ### denotes *p* < 0.001 vs. C; *** denotes *p* < 0.001 vs. 5-FU.

After skin damage induced by chemotherapy drugs, HaCaT keratinocytes divide and proliferate to cover the wound area. This process is known as “re-epithelialization” and is crucial for rapid wound closure. For this reason, we evaluated PPJE’s ability to promote skin regeneration processes by increasing the scratch repair and the speed of cell migration. We observed that PJJE (2.5–5 µg/mL) significantly enhanced cell migration speed compared to 5-FU-treated cells (*p* < 0.001 vs. 5-FU; Figure 6).

## 4. Discussion

Chemotherapy is the most widely used therapeutic approach for cancer treatment but its results are also associated with, sometimes, substantial adverse effects. The mechanism of action of many of these drugs, as well as radiotherapy, is the induction of tumor cells’ necrosis or apoptosis, with consequent ROS overproduction. Although oxidative stress, inflammation and apoptosis play a pivotal role as cellular mechanisms related to therapies for cancer treatment, to cause cancer cells elimination, they can also affect healthy cells. These side effects are particularly evident in tissue with a rapid turnover, such as skin, thus inducing effects that can significantly impair the quality of life in these patients. Therefore, control of oxidative stress and of the inflammatory response is of primary importance to avoid deleterious pathological conditions and to counteract anticancer treatment side effects.

Evidence has shown that many foods rich in polyphenols could be used for cancer prevention as adjuvants in chemotherapy treatments, but they could be used to also reduce cancer therapy side effects [41,42,43,44,45]. Pomegranate is a fruit rich in punicalagin, cyanidin 3,5-diglucoside and pelargonidin 3-glucoside [29], which have potent free-radical scavenging properties [46] and pomegranate has the potential to be an agent for cancer prevention related to the anti-inflammatory, pro-apoptotic and anti-proliferative activities of its constituents. In this study we focused our attention on the whole PPJE, reporting its protective effect on 5-FU-treated keratinocytes by: (i) reducing oxidative stress, (ii) reducing the inflammatory response, (iii) reducing apoptosis and (iv) promoting cell migration rate.

ROS are one of the principal mediators in tissue and cellular damage triggered by chemotherapy. In accordance with this, our data show that 5-FU induced a significant ROS release with respect to control cells and PPJE significantly inhibited ROS release in 5-FU-treated cells, at the three highest tested concentrations. This antioxidant effect of PPJE is in accordance with other evidence reporting the antioxidant effect of pomegranate on keratinocytes exposed to UV radiation [47] and in keratinocytes against H_2_O_2_-induced oxidative stress and cytotoxicity in human keratinocytes HaCaT [48]. If in normal conditions ROS production can be controlled by antioxidant mechanisms, including small-molecular-weight antioxidants and antioxidant enzyme systems protecting the organism from the damaging effects of oxidative stress, under conditions of severe oxidative stress, for example during the administration of certain drugs as chemotherapeutic agents, cellular endogenous antioxidant defense mechanisms may fail to control the excessive oxidative stress due to ROS overproduction. An oxidative stress condition is due to an imbalance between oxidant factors, such as ROS, and antioxidant factors, such as nuclear factor erythroid 2-like 2 (Nrf2). Nrf2 is a key regulator of the antioxidant response also in keratinocytes, being a redox-sensitive transcriptional regulator that controls the expression of antioxidant and detoxification enzymes such as HO-1 and NQO1 [49]. Our results indicated that PPJE significantly increased these Nrf-2-related antioxidant enzymes, in accordance with other experimental models reporting the protective effect of Nrf-2 pathway activation in keratinocytes during oxidative stress [50,51,52]. Evidence has shown that ROS activate NF-κB, thus amplifying the pro-inflammatory response [53,54]. Nitrotyrosine formation, due to the reaction between ROS and nitric oxide (NO), a pro-inflammatory mediator, is the stable product of the action of peroxynitrite on tyrosine-containing proteins. Nitrotyrosine residues can significantly impair the protein structure and alter its function, leading to potentially pathological results also in keratinocytes [55]. Our data indicated that PPJE was also able to significantly reduce nitrotyrosine formation thus also reducing the nitrosative stress response.

Proinflammatory cytokine production by epidermal keratinocytes plays a key role in skin inflammation [56]. Among these pro inflammatory cytokines, TNF-α has been implicated in the promotion of inflammatory reactions via the activation of cytokines IL-6 and IL-1β [56,57]. TNF-α released from keratinocytes would facilitate the subsequent influx of inflammatory cells on neighboring endothelial cells and it has been shown to promote the immune/inflammatory reactions via the activation and induction of other cytokines, such as IL-6 and IL-1β [58,59]. Therefore, inhibiting or limiting the production of these pro-inflammatory cytokines may help in the treatment of inflammation and dermatitis in cancer patients. PPJE treatment significantly inhibited TNF-α, IL-6 and IL-1β release, thus strongly contributing to the anti-inflammatory response. These data are in accordance with previous studies reporting the anti-inflammatory effect of pomegranate in different experimental models [60,61,62].

NF-κB is the most important transcriptional factor regulating inflammatory pathways. It also directly controls pro-inflammatory cytokine cellular production in keratinocytes suffering from 5-FU-induced dermatitis [15,55,63]. Our findings revealed that PPJE attenuated 5-FU-induced p65 NF-κB nuclear translocation, thereby reducing the expression of pro-inflammatory cytokines. Previous studies have reported that other agents, such as caffeic acid, saikosaponin A and palmitic acid, can reduce keratinocyte inflammation via NF-κB, TNF-α, IL-1β and IL-6 inhibition [55,56,64] thus highlighting the modulation of these factors as crucial in regulating 5-FU-induced skin side effects.

As a result of oxidative stress and inflammatory response onset, apoptosis occurs in 5-FU-treated keratinocytes relative to non-damaged control cells and PPJE significantly inhibited apoptosis in 5-FU-treated cells. The normal balance between pro-apoptotic members of the family (e.g., Bax) and anti-apoptotic components (e.g., Bcl-2) might also be disrupted by the administration of chemotherapy in healthy cells. PPJE was able to reduce Bax expression and to increase the anti-apoptotic protein Bcl-2, thus contributing to protecting keratinocytes from cell death. Our results are in accordance with other studies reporting the protective effect of pomegranate in protecting normal cells from various stimuli-induced toxicities and apoptosis [65].

Keratinocytes play a fundamental role in maintaining the integrity of the epidermal barrier [66], also taking part in skin repair processes and for these reasons their homeostasis is of primary importance. 5-FU treatment induced a significant reduction in cell migration rate, as assessed in wound healing assay, but PPJE induced a significant increase in cell migration speed, thus promoting the skin damage resolution. The obtained results highlight the potential use of PPJE in counteracting chemotherapy-induced side effects, also because this potential can be exerted with topical use. Thus, PPJE would not interfere in reducing the pro-oxidant, pro-inflammatory and pro-apoptotic effect of the therapy on the cancer cells.

## 5. Conclusions

The results of this study support the antioxidant and anti-inflammatory effects of pomegranate in a course of chemotherapeutic treatment. These effects, together with the anti-apoptotic properties and with the ability to promote wound healing, highlight the potential of using PPJE as a topic onconutraceutical treatment for 5-FU-induced skin damage. This study highlights the potential of pomegranate which appears to be a promising strategy not only in cancer prevention, but also in the management of chemotherapy side effects.

## Data Availability

The data presented in this study are available on request from the corresponding author.
